# Discovery of Pyrazolo[4,3-*c*]quinolines Derivatives as Potential Anti-Inflammatory Agents through Inhibiting of NO Production

**DOI:** 10.3390/molecules23051036

**Published:** 2018-04-28

**Authors:** Chih-Hua Tseng, Chun-Wei Tung, Shin-I Peng, Yeh-Long Chen, Cherng-Chyi Tzeng, Chih-Mei Cheng

**Affiliations:** 1 School of Pharmacy, College of Pharmacy, Kaohsiung Medical University, Kaohsiung City 807, Taiwan; cwtung@kmu.edu.tw; 2 Department of Fragrance and Cosmetic Science, College of Pharmacy, Kaohsiung Medical University, Kaohsiung City 807, Taiwan; 3 Center for Infectious Disease and Cancer Research, Kaohsiung Medical University, Kaohsiung City 807, Taiwan; 4 Department of Medical Research, Kaohsiung Medical University Hospital, Kaohsiung City 807, Taiwan; 5 Ph.D. Program in Toxicology, Kaohsiung Medical University, Kaohsiung City 807, Taiwan; 6 Department of Medicinal and Applied Chemistry, College of Life Science, Kaohsiung Medical University, Kaohsiung City 807, Taiwan; andrea6599@gmail.com (S.-I.P.); yeloch@kmu.edu.tw (Y.-L.C.); tzengch@kmu.edu.tw (C.-C.T.); 7 Department of Biomedical Science and Environmental Biology, College of Life Science, Kaohsiung Medical University, Kaohsiung City 807, Taiwan

**Keywords:** pyrazolo[4,3-*c*]quinolines, nitric oxide, anti-inflammatory activity

## Abstract

The synthesis and anti-inflammatory effects of certain pyrazolo[4,3-*c*]quinoline derivatives **2a**–**2r** are described. The anti-inflammatory activities of these derivatives were evaluated by means of inhibiting nitric oxide (NO) production in lipopolysaccharide (LPS)-induced RAW 264.7 cells. Among them, 3-amino-4-(4-hydroxyphenylamino)-1*H*-pyrazolo[4,3-*c*]-quinoline (**2i**) and 4-(3-amino-1*H*-pyrazolo[4,3-*c*]quinolin-4-ylamino)benzoic acid (**2m**) exhibited significant inhibition of LPS-stimulated NO production with a potency approximately equal to that of the positive control, 1400 W. Important structure features were analyzed by quantitative structure–activity relationship (QSAR) analysis to give better insights into the structure determinants for predicting the inhibitory effects on the accumulation of nitric oxide for RAW 264.7 cells in response to LPS. In addition, our results indicated that their anti-inflammatory effects involve the inhibition of inducible nitric oxide synthase (iNOS) and cyclooxygenase 2 (COX-2) protein expression. Further studies on the structural optimization are ongoing.

## 1. Introduction

Nitric oxide (NO), a gaseous free radical, is an important signaling molecule that is involved in a wide range of pathophysiological responses such as inflammation, carcinogenesis, and vasodilation [1,2,3]. In mammals, NO is produced from the enzymatic oxidation of l-arginine by three types of nitric oxide synthase (NOS): neuronal NOS (nNOS), endothelial NOS (eNOS) and inducible NOS (iNOS) [4]. nNOS and eNOS which produced low concentrations of NO are constitutive and Ca^2+^-dependent enzymes [5]. iNOS is ubiquitous, Ca^2+^-independent, and frequently overexpressed by pro-inflammatory and/or carcinogenic stimuli such as interleukin-1β (IL–1β), tumor necrosis factor-α (TNF-α) and lipopolysaccharide (LPS) in macrophages, endothelial cells and smooth muscle cells [6]. Once expressed at high levels, and iNOS is essentially unregulated resulting in local tissue damage and many inflammatory diseases including rheumatoid arthritis, osteoarthritis, inflammatory bowel disease and multiple sclerosis. Therefore, the control of NO production through iNOS inhibition provides a promising strategy for the development of potential anti-inflammatory agents.

Many efforts have been devoted to the discovery of inhibitor of iNOS for the past few years [7,8,9,10,11,12,13]. We have also reported that furo[3,2:3,4]naphtha[1,2-*d*] imidazole derivatives represent a novel class of inhibitors of iNOS [14]. Pyrazolo[4,3-*c*]quinolines has recently received much attention for its anti-inflammatory, anti-cancer and β-glucuronidase inhibitory activities [15,16].

This study is aimed to demonstrate the anti-inflammatory effects of pyrazolo[4,3-*c*]quinoline derivatives which exhibited potent inhibitory activities on the LPS-stimulated NO production and iNOS expression in RAW 264.7 cells.

## 2. Chemistry

**Scheme 1:**
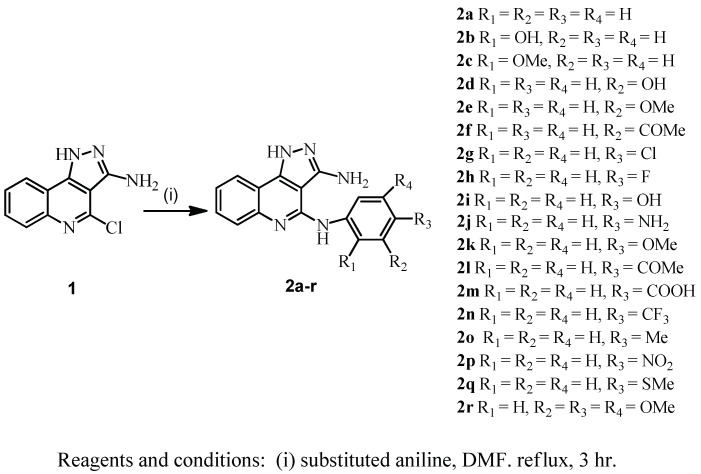
Synthesis of pyrazolo[4,3-*c*]quinolines derivatives **2a**–**r**.

According to previously reported procedures [16,17], 4-chloro-1*H*-pyrazolo[4,3-*c*]quinolin-3-amine (**1**) was reacted with substituted anilines to give 3-amino-4-(substituted phenylamino)-1*H*-pyrazolo[4,3-*c*]-quinoline derivatives **2c, 2e, 2f, 2q** and **2r** as depicted in Scheme 1. Preparation of compounds **2a, 2b, 2d,** and **2g**–**2p** had been described in our previous report [16].

## 3. Results and Discussion

### 3.1. Lipopolysaccharide (LPS)-Induced Nitric Oxide Production

Treatment of Raw 264.7 cells with LPS (0.1 μg/mL) for 24 h induced NO production which were assessed by measuring the accumulation of nitrite, a stable metabolite of NO, in the media based on Griess reaction [18]. The inhibition of LPS-stimulated NO production and cytotoxicity of pyrazolo[4,3-*c*]quinoline derivatives are summarized in Table 1. 3-Amino-4-phenylamino-1*H*-pyrazolo[4,3-*c*]quinoline (**2a**) displayed a significant inhibitory activity on the LPS-induced NO production with an IC_50_ value of 0.39 μM. However, compound **2a** was also highly cytotoxic, in which the survival rate of RAW 264.7 cells was only 9% at a drug concentration of 10 μM. *Ortho*-substitution of the electron-donating group on the phenyl ring, such as OH (compound **2b**) and OMe (compound **2c**), slightly decreased the inhibitory activity on the LPS-induced NO production and also decreased cytotoxicity. The position of substitution play an important role in which substitution at *para*-position is usually more favorable than its *ortho*- or *meta*- counterpart in the inhibitory activity on the LPS-induced NO production. For the OH-substituted pyrazolo[4,3-*c*]quinolones, **2i** (*para*, IC_50_ value of 0.19 μM) > **2b** (*ortho*, IC_50_ value of 0.42 μM) > **2d** (*meta*, IC_50_ value of 0.77 μM). For the OMe-derivatives, **2k** (*para*; IC_50_ value of 0.48 μM) equal to **2c** (*ortho*; IC_50_ value of 0.49 μM) > **2e** (*meta*; IC_50_ value of 0.77 μM) while the COMe-derivatives, **2l** (*para*; IC_50_ value of 0.29 μM) equal to **2f** (*meta*; IC_50_ value of 0.29 μM). Various substituted groups have been introduced on the *para*-position of the phenyl ring. Among them, both 3-amino-4-(*para*-hydroxyphenylamino)-1*H*-pyrazolo[4,3-*c*]quinoline (**2i**) and its *para*-carboxylic acid counterpart **2m** were able to suppress LPS-induced NO production with an IC50 value of 0.19 and 0.22 μM, respectively, which are equally active to that of the positive *N*-(3-(aminomethyl)benzyl)acetamidine (1400 W) [19].

### 3.2. Nitric Oxide (NO)-Scavenging Effects

**Figure 1:**
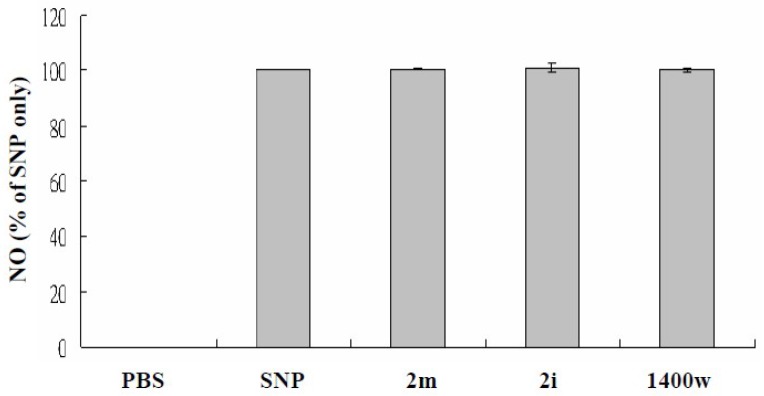
NO-scavenging effects of 1400W, compounds **2i** and **2m**. SNP solution (2.5 mM) in PBS was incubated alone or with tested compound (10 μM) or 1400W (10 μM) in the presence of light at room temperature for 60 min.

NO-scavenging effects of pyrazolo[4,3-*c*]quinolines **2i** and **2m** were measured according to the reported method [20]. Sodium nitroprusside (SNP) (2.5 mM) was incubated alone or in combination with tested compound (10 μM) or 1400W(10 μM) in light at room temperature for 60 min. Results of Figure 1 indicated the co-incubation of SNP with compounds **2i** and **2m** or 1400W did not affect the levels of nitrite.

### 3.3. Lipopolysaccharide (LPS)-Induced iNOS Protein and mRNA Expression

**Figure 2:**
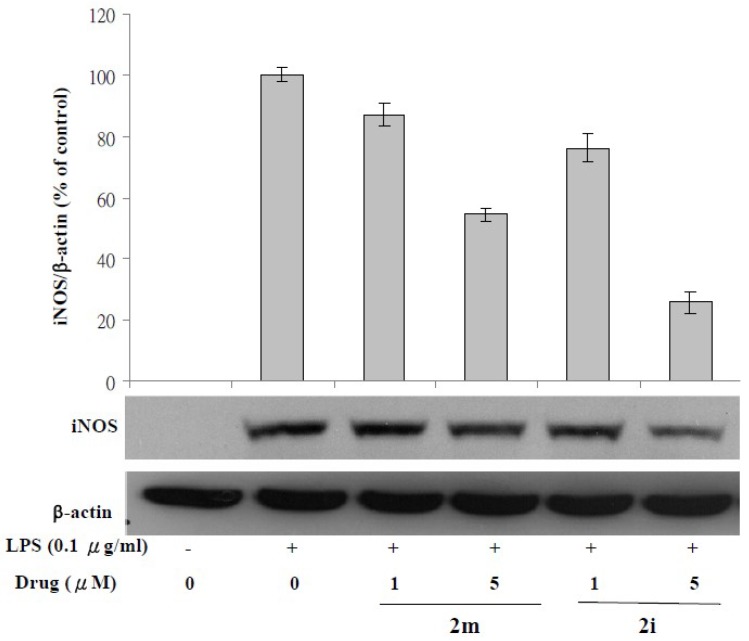
Inhibition of iNOS expression by **2i** and **2m** in LPS-stimulated RAW 264.7 cells. Cells (5 × 10^5^ cells/well) were pretreated with the indicated concentrations of tested compounds for 2 h before incubation with 0.1 μg/mL LPS for 24 h. Cell lysates were then prepared and western blots were performed using an antibody specific for murine iNOS.

Many studies have demonstrated that induction of iNOS produces a large amount of NO during endotoxemia and under inflammatory conditions. Therefore, drugs that inhibit iNOS expression and/or enzyme activity, resulting in decreased NO generation, may be beneficial in treating diseases caused by an overproduction of NO [21,22]. To clarify the possible mechanism of involved in **2i** and **2m**, we examined the inhibitory effects of two compounds, on the protein expression of iNOS in Raw 264.7 cells. Results from Figure 2 indicated the unstimulated cells expressed negligible levels of iNOS protein whereas LPS-induced cells exhibited higher protein levels. Compound **2i** exhibited more potent inhibitory activities than **2m**. 

**Figure 3:**
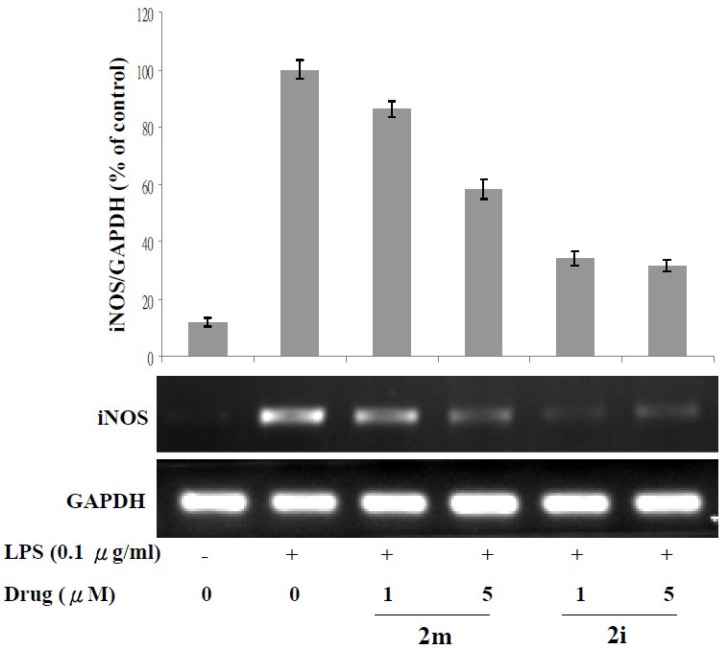
Inhibitory effects of compound **2i** and **2m** on LPS-induced iNOS mRNA expression. Cells (5 × 10^5^ cells/well) were pretreated with the indicated concentrations of tested compounds for 2 h before incubation with 0.1 μg/mL LPS for 9 h. Total RNA was prepared for RT-PCR analysis of iNOS mRNA expression in LPS-stimulated RAW 264.7 cells. GAPDH were used as internal controls for the RT-PCR assays3.4. Inhibition of LPS-inducible COX-2 expression.

Two compounds were capable of inhibiting LPS-induced iNOS protein expression at a dose-dependent manner. These results are in agreement with their inhibitory effects on NO production (Table 1). Thus, the results of NO inhibition may be directly mediated by the down regulation of iNOS protein expression. To assess the effect of **2i** and **2m** on iNOS mRNA expression, we measured mRNA levels by Northern blot analysis. The expression of iNOS mRNA was hardly detectable in unstimulated cells. However, RAW 264.7 cells expressed high levels of iNOS mRNA when stimulated with LPS (0.1 μg/mL) for 24 h. Furthermore, compound **2i** and **2m** inhibited the LPS-stimulated iNOS mRNA production in a dose-dependent manner (Figure 3). RT-PCR and western blot results revealed that iNOS expression by **2i** and **2m** were in parallel with the comparable inhibition of NO production.

**Figure 4:**
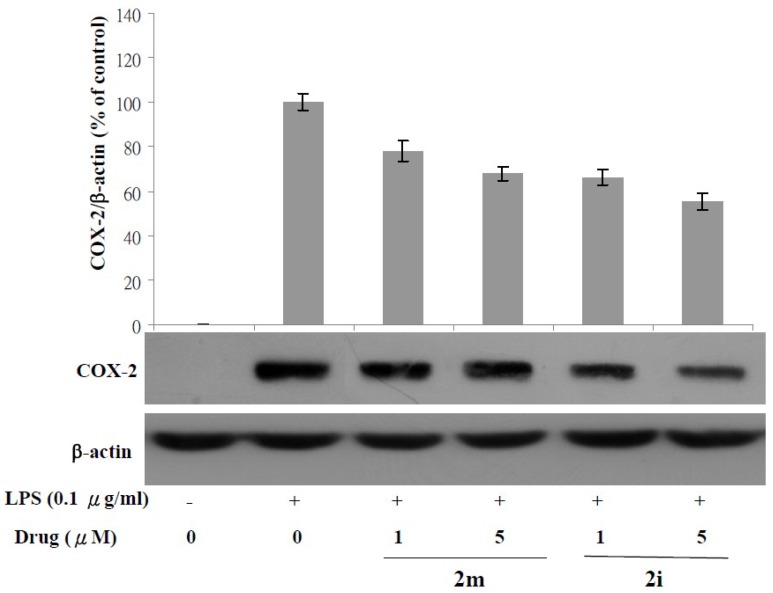
Inhibition of COX-2 expression by compound **2i** and **2m** in LPS-stimulated RAW 264.7 cells. Cells (5 × 10^5^ cells/well) were pretreated with the indicated concentrations of tested compounds for 2 h before incubation with 0.1 μg/mL LPS for 24 h. Cell lysates were then prepared and western blots were performed using an antibody specific for murine COX-2.

Cyclooxygenase 2 (COX-2) is induced by LPS, certain serum factors, cytokines, and growth factors, and is a predominant cyclooxygenase at sites of inflammation. A major advance in developing Cox-2 inhibitors is that they may be used in prevention or treatment of the inflammatory disorder that is associated with the induction of this enzyme [23]. Recent findings suggest that COX-2 may play an important role in the pathogenesis of diseases such as colon carcinoma, Alzheimer’s disease, and hypertension [24,25,26]. We further evaluated the effect of **2i** and **2m** on LPS-inducible COX-2 protein expression in macrophages. The expression of COX-2 protein was monitored in RAW 264.7 cells exposed to LPS (0.1 μg/mL) for 24 h. Compounds **2i** and **2m** effectively suppressed the induction of COX-2 protein expression by LPS (Figure 4).

### 3.4. Quantitative Structure-Activity Relationship (QSAR)

In this study, we performed quantitative structure-activity relationship (QSAR) study to further investigate the relationship between the structure descriptors and inhibitory effects on the accumulation of nitric oxide for RAW 264.7 cells in response to LPS. The pIC_50_ values representing the negative logarithm of IC_50_ values were utilized for developing QSAR models. First, a total of 18 chemicals listed in Table 2 were randomly divided into training and test datasets consisting of 12 (**2a, 2b, 2g, 2i, 2j, 2l, 2m, 2n, 2o, 2p, 2q** and **2r**) and six (**2c**, **2d**, **2e**, **2f**, **2h** and **2k**) chemicals, respectively. Subsequently, chemicals were transformed into 2325-dimentional vectors representing structure features. A sequential feature selection algorithms developed by our group [27,28,29] were then applied to simultaneously select a minimum number of relevant descriptors maximizing the R^2^ performance and develop multiple regression models for QSAR analysis. Only four most important features were considered due to the small number of chemicals in the dataset. Based on the dataset, the model is expected to be useful for chemicals within the IC_50_ range from 0.19 to 0.92 μM.

A high performance of *R*^2^ = 0.97, *R*^2^_adj_ = 0.96, RMSE = 0.058, and *Q*^2^ = 0.91 was obtained for the constructed QSAR model that could well explain the variation of IC_50_ values. Table 2 shows the QSAR model and four important descriptors of SsNH2, SHBint9, nHBAcc and AATSC4m. The observed and predicted pIC_50_ values of the training dataset are shown in Table 3. The atom type electrotopological state descriptors of SsNH2 and SHBint9 represents the count of atom-type E-State for -SnH2- and the sum of E-State descriptors of strength for potential hydrogen bonds of path length 9, respectively [30,31,32]. The nHBAcc is a count of hydrogen bond acceptors using CDK HBondAcceptorCountDescriptor algorithm [33]. The autocorrelation descriptor of AATSC4m denotes the average centered Broto-Moreau autocorrelation - lag 4/weighted by mass [34].

Among the important descriptors, SsNH2 negatively correlate with pIC_50_ value with a high correlation coefficient of −0.77. Although the other three descriptors of SHBint9, nHBAcc and AATSC4m show no direct correlation to pIC_50_ with correlation coefficients of 0.03, 0.02 and −0.33, respectively, their importance has been shown by a 29% improvement on the R^2^ performance via the inclusion of the three descriptors. 

**Figure 5:**
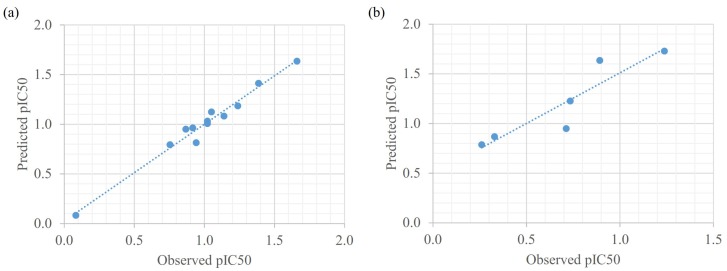
Scatter plots of the QSAR analysis for (**a**) training dataset and (**b**) test dataset.

The test dataset consisting of six chemicals was utilized to further evaluate the constructed QSAR model. While the predicted errors are relatively large due to the inclusion of three chemicals with low pIC_50_ values, the predicted pIC_50_ values are well correlated with the observed pIC_50_ values with a correlation coefficient of 0.92 as shown in Table 4. Figure 5 shows the scatter plots for comparison of observed and predicted pIC_50_ values of training and test datasets. The model could be further tested and improved when more data are available.

## 4. Experimental

### 4.1. General Information

Melting points were determined on an IA9100 melting point apparatus Electrothermal (Dubuque, IA, USA) and are uncorrected. Nuclear magnetic resonance (^1^H) spectra were recorded on a Unity-400 spectrometer (Varian, Palo Alto, CA, USA) (Supplementary materials). Chemical shifts were expressed in parts per million (δ) with tetramethylsilane (TMS) as an internal standard. Thin-layer chromatography was performed on silica gel 60 F-254 plates purchased from E. Merck and Co. (Darmstadt, Germany). The elemental analyses were performed in the Instrument Center of National Science Council at National Cheng-Kung University and National Chung-Hsing University using a CHN-O Rapid EA system (Heraeus, Waltham, MA, USA) and all values are within ±0.4% of the theoretical compositions.

#### 4.1.1. 3-Amino-4-(2-methoxyphenylamino)-1*H*-pyrazolo[4,3-*c*]quinoline (**2c**)

Yield 69%. Mp.: 210–211 °C. ^1^H-NMR (400 MHz, DMSO-*d_6_*): 5.22 (s, 2H, NH_2_), 6.96–7.08 (m, 3H, Ar-H), 7.29–7.32 (m, 1H, Ar-H), 7.50–7.54 (m, 1H, Ar-H), 7.69 (d, 1H, *J* = 8.0 Hz, Ar-H), 8.06 (d, 1H, *J* = 8.0 Hz, Ar-H), 8.57 (s, 1H, NH), 8.99 (d, 1H, *J* = 7.2 Hz, Ar-H), 13.03 (s, 1H, NH) (Supplementary materials Figure S1). Anal. calcd for C_17_H_15_N_5_O**∙**0.45H_2_O : C 65.13, H 5.12, N 22.35; found: C 65.31, H 5.03, N 21.99.

#### 4.1.2. 3-Amino-4-(3-methoxyphenylamino)-1*H*-pyrazolo[4,3-*c*]quinoline (**2e**)

Yield 76%. Mp.: 184–186 °C. ^1^H-NMR (400 MHz, DMSO-*d_6_*): 3.81 (s, 3H, OMe), 5.63 (br s, 2H, NH_2_), 6.58–6.61 (m, 1H, Ar-H), 7.22–7.26 (m, 1H, Ar-H), 7.29–7.33 (m, 1H, Ar-H), 7.44 (d, 1H, *J* = 8.0 Hz, Ar-H), 7.50–7.54 (m, 1H, Ar-H), 7.65 (d, 1H, *J* = 8.0 Hz, Ar-H), 7.90 (s, 1H, Ar-H), 8.07 (d, 1H, *J* = 7.6 Hz, Ar-H), 8.24 (s, 1H, NH), 12.95 (br s, 1H, NH) (Supplementary materials Figure S2). Anal. calcd for C_17_H_15_N_5_O : C 66.87, H 4.95, N 22.94; found: C 66.64, H 4.98, N 22.68.

#### 4.1.3. 3-Amino-4-(3-acetylphenylamino)-1*H*-pyrazolo[4,3-*c*]quinoline (**2f**)

Yield 84%. Mp.: >380 °C. ^1^H-NMR (400 MHz, DMSO-*d_6_*): 2.63 (s, 3H, Me), 7.45–7.49 (m, 1H, Ar-H), 7.57–7.60 (m, 1H, Ar-H), 7.68–7.72 (m, 1H, Ar-H), 7.76 (d, 1H, *J* = 8.4 Hz, Ar-H), 7.89 (d, 1H, *J* = 8.8 Hz, Ar-H), 7.97 (d, 1H, *J* = 7.6 Hz, Ar-H), 8.17−8.20 (m, 2H, Ar-H), 11.05 (br s, 1H, NH), 11.85 (br s, 1H, NH) (Supplementary materials Figure S3). Anal. calcd for C_18_H_15_N_5_O**∙**0.5H_2_O : C 66.23, H 4.95, N 21.46; found: C 66.31, H 4.66, N 21.26.

#### 4.1.4. 3-Amino-4-(4-methylthiophenylamino)-1*H*-pyrazolo[4,3-*c*]quinoline (**2q**)

Yield 80%. Mp.: 269–271 °C. ^1^H-NMR (400 MHz, DMSO-*d_6_*): 2.48 (s, 3H, SMe), 5.63 (br s, 2H, NH_2_), 7.29 (d, 1H, *J* = 8.8 Hz, Ar-H), 7.48–7.51 (m, 1H, Ar-H), 7.61 (d, 1H, *J* = 8.0 Hz, Ar-H), 7.95 (d, 1H, *J* = 8.4 Hz, Ar-H), 8.05 (d, 1H, *J* = 7.6 Hz, Ar-H), 8.24 (br s, 1H, NH), 12.91 (br s, 1H, NH) (Supplementary materials Figure S4). Anal. calcd for C_17_H_15_N_5_S**∙**0.25H_2_O: C 62.64, H 4.80, N 21.49; found: C 62.66, H 4.72, N 21.14.

#### 4.1.5. 3-Amino-4-(3,4,5-trimethoxyphenylamino)-1*H*-pyrazolo[4,3-*c*]quinoline (**2r**)

Yield 71%. Mp.: 161–162 °C. ^1^H-NMR (400 MHz, DMSO-*d_6_*): 3.66 (s, 3H, OMe), 3.84 (s, 6H, OMe × 2) 5.66 (br s, 2H, NH_2_), 7.28–7.32 (m, 1H, Ar-H), 7.50–7.52 (m, 3H, Ar-H), 7.65 (d, 1H, *J* = 8.0 Hz, Ar-H), 8.06 (d, 1H, *J* = 6.8 Hz, Ar-H), 8.12 (br s, 1H, NH), 12.93 (br s, 1H, NH) (Supplementary materials Figure S5). Anal. calcd for C_19_H_19_N_5_O_3_**∙**1.0H_2_O : C 59.51, H 5.53, N 18.27; found: C 59.62, H 5.50, N 18.20.

### 4.2. Cell Culture

Raw 264.7 cells, purchased from Bioresource Collection and Research Center, Taiwan, were cultured in DMEM, supplemented with 5% fetal bovine serum, 100 units/mL of penicillin, 100 μg/mL of streptomycin, 2 mM l-glutamine, and 1 mM non-essential amino acids in a 10-cm plate at a density of 1 × 10^6^ cells/mL, at 37 °C in a humidified atmosphere containing 5% CO_2_.

#### 4.2.1. Nitrite Measurement

Nitrite was measured by adding 100 μL of the Griess reagent (1% sulfanilamide and 0.1% *N*-(1-naphthyl)ethylenediamine dihydrochloride in 5% phosphoric acid) to 100 μL of medium for 5 min. The optical density at 550 nm (OD_550_) was measured with a microplate reader. Concentrations were calculated by comparison with the OD_550_ of a standard solution of sodium nitrite prepared in culture medium.

#### 4.2.2. Cell Viability

To evaluate whether the suppressive effect of compounds on NO production is related to cell viability, Raw 264.7 cells were treated with LPS for 24 h, and washed with 500 μL of phosphate buffer solution (PBS) before resuspended in 1 mL of culture medium. Cell viability tests were run in parallel with NO production assays with an initial cell density of 5 × 10^3^ cells/well in the presence or absence of test compounds at 10 μM or 0.1% DMSO as control. Cell viability was determined after 24 h at 37 °C in a humidified CO_2_ (5%) atmosphere by the (2,3-bis[2-methyloxy-4-nitro-5-sulfophenyl]-2*H*- tetrazolium-5-carboxanilide) (XTT) method [35].

#### 4.2.3. NO-Scavenging Activity

To understand the inhibitory effects of compounds **2i** and **2m** on NO production, sodium nitroprusside (SNP) was freshly prepared at 5 mM in PBS was incubated alone or in combination with 10 lM of the different compounds. SNP is an inorganic complex where NO is found as NO^+^ and light irradiation is necessary for the release of NO. Therefore, incubation mixtures were incubated in light at room temperature, and nitrite levels were determined after 60 min by Griess reaction.

#### 4.2.4. Western Blotting

The effects of compounds **2i** and **2m** on iNOS and COX-2 protein expression in LPS stimulated Raw 264.7 cell were assessed by western blot. Briefly, Raw 264.7 cells were pretreated with tested compound for 2 h before stimulation in the presence or absence of LPS (0.1 μg/mL). Cells were harvested and lysed with RIPA lysis buffer (50 mM Tris–Cl, pH 8.0, 150 mM NaCl, 0.1% SDS, 0.5% NP-40, 1% sodiumdeoxycholate, 1 mM phenylmethyl-sulfonylfluoride, 1 mM EDTA, no added proteinase) at 24 h after treatment. The total protein concentration in the cell lysates was measured according to the method described by Bradford [36]. Equal amounts (15 μg) of protein were loaded and separated by using 8% SDS–PAGE and analyzed by western blot (ECL Amersham, Bensheim, Germany). The relative expression of proteins was quantified densitometrically using the software LabWorks 4.5 and was normalized againstβ-actin.

#### 4.2.5. RNA Extraction and Determination of iNOS mRNA Expression

Total RNA was extracted by Trizol-Reagent (Invirogen, Carlsbad, CA, USA) according to the manufacturer’s manual. One microgram of RNA was used for the reverse transcription of first strand cDNA using the oligo dT primers. Five microliters of cDNA were used for the amplification of iNOS and GAPDH (glyceraldehyde 3-phosphate dehydrogenase) mRNA expression. The primers for rat iNOS are as follows, forward: 5′-ACT GCG TCG CTT CAT TAG GT-3′ and reverse: 5′-TAG GCA AGC GCT TTA CCA CT-3′. The primers for rat glyceraldehydes 3-phosphate dehydrogenase are as follows, forward: 5′-ATG GCA CAG TCA AGG CTG AGA-3′ reverse: 5′-AGA CGC CAG TAG ACT CCA CGA C-3′. The amplicons were electrophoresed on a 3% agarose gel and band intensities were measured using a spectrophotometer (Thermo Fisher Scientific, Waltham, MA, USA) and normalized against GAPDH.

#### 4.2.6. Quantitative Structure-Activity Relationship (QSAR)

For QSAR analyses, the PaDEL-Descriptor [37] software was firstly applied to generate 2325 structure features of 1D and 2D descriptors and PubChem fingerprints for each chemical. The calculation mainly bases on the Chemistry Development Kit [33] with some additional descriptors and fingerprints. Sequential feature selection algorithms developed by our group [27,28,29] were subsequently applied to simultaneously identify important features and build multiple regression models. Given a maximum number *n* of selected features, the descriptor with highest R^2^ performance was iteratively appended into the multiple regression model for *n* runs. The descriptor set with the highest R^2^ performance is utilized to construct the final multiple regression model for QSAR analysis. Four measurements of *R*^2^, *R*_adj_^2^, root mean squared error (RMSE), and leave-one-out cross-validated *Q*^2^ were applied to evaluate the performance of the QSAR model.

## 5. Conclusions

We have synthesized and evaluated certain pyrazolo[4,3-*c*]quinoline derivatives for their inhibitory activities of LPS-stimulated NO production and iNOS expression in RAW 264.7 cells. These compounds were found to have potent inhibitory activities of LPS-stimulated NO production with the IC_50_ in a submicromolar concentration. Among them, 3-amino-4-(4-hydroxyphenylamino)-1*H*-pyrazolo[4,3-*c*]-quinoline (**2i**) and 4-(3-amino-1*H*-pyrazolo[4,3-*c*]quinolin-4-ylamino)benzoic acid (**2m**) exhibited significant inhibition of LPS-stimulated NO production with a potency approximately equal to that of the positive control, 1400W. Furthermore, our results indicated that the possible mechanism by which their anti-inflammatory effects involve the inhibition of iNOS and COX-2 protein expression. Further studies on the structural optimization are ongoing.

## Figures and Tables

**Table 1:** The inhibitory effects of pyrazolo[4,3-*c*]quinoline derivatives on the accumulation of nitric oxide in the culture media of RAW 264.7 cells in response to LPS.

Compound	^a^ Percent Inhibition of NO Production at 10 μM	^a^ IC_50_ ( μM)	^a^ Cell Survival Rate at 10 μM
**2a**	81.66 ± 0.99	0.39 ± 0.08	9.09 ± 0.24
**2b**	103.44 ± 6.55	0.42 ± 0.11	53.48 ± 1.53
**2c**	82.23 ± 6.49	0.49 ± 0.14	47.31 ± 1.67
**2d**	93.52 ± 7.57	0.77 ± 0.23	81.70 ± 0.09
**2e**	101.43 ± 1.31	0.72 ± 0.18	94.32 ± 0.49
**2f**	99.14 ± 2.16	0.29 ± 0.06	94.37 ± 0.36
**2g**	103.72 ± 6.71	0.32 ± 0.05	81.61 ± 0.14
**2h**	94.84 ± 9.47	0.41 ± 0.07	89.99 ± 0.02
**2i**	101.72 ± 2.63	0.19 ± 0.05	87.84 ± 0.15
**2j**	95.13 ± 3.75	0.92 ± 0.05	90.26 ± 0.05
**2k**	90.83 ± 11.94	0.48 ± 0.14	90.57 ± 0.03
**2l**	102.01 ± 6.49	0.29 ± 0.08	87.80 ± 0.11
**2m**	87.68 ± 9.93	0.22 ± 0.07	85.42 ± 0.77
**2n**	106.88 ± 2.48	0.36 ± 0.09	69.14 ± 0.62
**2o**	100.03 ± 3.02	0.47 ± 0.07	84.53 ± 0.04
**2p**	89.11 ± 0.86	0.40 ± 0.09	90.86 ± 0.32
**2q**	102.29 ± 7.01	0.36 ± 0.08	89.62 ± 0.05
**2r**	107.16 ± 2.58	0.35 ± 0.06	85.24 ± 0.04
1400w ^b^	102.15 ± 2.16	0.20 ± 0.02	99.54 ± 2.51

^a^ Values are means ± S.D. of at least three separate experiments. ^b^ *N*-[[3-(Aminomethyl)-phenyl]methyl]-ethanimidamide dihydrochloride (1400W) was used as a positive control.

**Table 2:** The QSAR analysis result for pIC_50_ values.

	Coefficient	Estimate Std. Error	t-Value	Pr (>|t|)	
(Intercept)	1.258	0.196	6.42	3.60 × 10^−4^	***
SsNH2	−0.262	0.017	−15.16	1.30 × 10^−6^	***
SHBint9	0.049	0.005	9.21	3.70 × 10^−5^	***
nHBAcc	0.226	0.038	5.96	5.60 × 10^−4^	***
AATSC4m	−0.037	0.011	−3.46	1.06 × 10^−2^	*

*** *p* < 0.001, ** *p* < 0.01, * *p* < 0.05.

**Table 3:** The comparison of observed and predicted values for the training dataset.

Compound	Observed pIC_50_	Predicted pIC_50_	Error
**2j**	0.083	0.083	0.000
**2o**	0.755	0.794	−0.039
**2b**	0.868	0.950	−0.083
**2p**	0.916	0.963	−0.047
**2a**	0.942	0.814	0.128
**2n**	1.022	1.032	−0.010
**2q**	1.022	1.007	0.015
**2r**	1.050	1.125	−0.075
**2g**	1.139	1.082	0.058
**2l**	1.238	1.185	0.053
**2m**	1.386	1.413	−0.027
**2i**	1.661	1.636	0.025

**Table 4:** The comparison of observed and predicted values for the test dataset.

Compound	Observed pIC_50_	Predicted pIC_50_	Error
**2d**	0.261	0.786	−0.524
**2e**	0.329	0.868	−0.540
**2c**	0.713	0.949	−0.236
**2k**	0.734	1.227	−0.493
**2h**	0.892	1.635	−0.743
**2f**	1.238	1.729	−0.491

## Supplementary Materials

Supplementary materials are available online.

## References

[1] Singer I.I., Kawka D.W., Scott S., Weidner J.R., Mumford R.A., Riehl T.E., Stenson W.F. (1996). Expression of inducible nitric oxide synthase and nitrotyrosine in colonic epithelium in inflammatory bowel disease. Gastroenterology.

[2] Nguyen T., Brunson D., Crespi C.L., Penman B.W., Wishnok J.S., Tannenbaum S.R. (1992). DNA damage and mutation in human cells exposed to nitric oxide in vitro. Proc. Natl. Acad. Sci. USA.

[3] Rees D.D., Palmer R.M.J., Moncada S. (1989). Role of Endothelium-derived nitric oxide in the regulation of blood pressure. Proc. Natl. Acad. Sci. USA.

[4] Marletta M.A. (1993). Nitric oxide synthase structure and mechanism. J. Biol. Chem..

[5] Grisham M.B., Jourd H.D., Wink D.A.I. (1999). Physiological chemistry of nitric oxide and its metabolites: Implications in inflammation. Am. J. Physiol. Gastrointest. Liver Physiol..

[6] Morikawa A., Koide N., Kato Y., Sugiyama T., Chakravortty D., Yoshida T., Yokochi T. (2000). Augmentation of nitric oxide production by gamma interferon in a mouse vascular endothelial cell line and its modulation by tumor necrosis factor alpha and lipopolysaccharide. Infect. Immun..

[7] Bach D.H., Liu J.Y., Kim W.K., Hong J.Y., Park S.H., Kim D., Qin S.N., Luu T.T., Park H.J., Xu Y.N. (2017). Synthesis and biological activity of new phthalimides as potential anti-inflammatory agents. Bioorg. Med. Chem..

[8] Ma L., Xie C., Ma Y., Liu J., Xiang M., Ye X., Zheng H., Chen Z., Xu Q., Chen T. (2011). Synthesis and biological evaluation of novel 5-benzylidenethiazolidine-2,4-dione derivatives for the treatment of inflammatory diseases. J. Med. Chem..

[9] Ma L., He L., Lei L., Liang X., Lei K., Zhang R., Yang Z., Chen L. (2015). Synthesis and biological evaluation of 5-nitropyrimidine-2,4-dione analogues as inhibitors of nitric oxide and iNOS activity. Chem. Biol. Drug Des..

[10] Ma L., Pei H., Lei L., He L., Chen J., Liang X., Peng A., Ye H., Xiang M., Chen L. (2015). Structural exploration, synthesis and pharmacological evaluation of novel 5-benzylidenethiazolidine-2,4-dione derivatives as iNOS inhibitors against inflammatory diseases. Eur. J. Med. Chem..

[11] Mahapatra D.K., Bharti S.K., Asata V. (2017). Chalcone derivatives: Anti-inflammatory potential and molecular targets perspectives. Curr. Top. Med. Chem..

[12] Bluhm U., Boucher J.L., Buss U., Clement B., Friedrich F., Girreser U., Heber D., Lam T., Lepoivre M., Rostaie-Gerylow M. (2009). Synthesis and evaluation of pyrido[1,2-*a*]pyrimidines as inhibitors of nitric oxide synthases. Eur. J. Med. Chem..

[13] Hu X.L., Lin J., Lv X.Y., Feng J.H., Zhang X.Q., Wang H., Ye W.C. (2018). Synthesis and biological evaluation of clovamide analogues as potent anti-neuroinflammatory agents in vitro and in vivo. Eur. J. Med. Chem..

[14] Tseng C.H., Lin C.S., Shih P.K., Tsao L.T., Wang J.P., Cheng C.M., Tzeng C.C., Chen Y.L. (2009). Furo[3,2:3,4]naphtho[1,2-*d*]imidazole derivatives as potential inhibitors of inflammatory factors in sepsis. Bioorg. Med. Chem..

[15] Mekheimer R. (1994). A convenient synthesis of new substituted pyrazolo[4,3-*c*]quinolines with potentialantiinflammatory activity. Pharmazie.

[16] Cheng K.W., Tseng C.H., Yang C.N., Tzeng C.C., Cheng T.C., Leu Y.L., Chuang Y.C., Wang J.Y., Lu Y.C., Chen Y.L. (2017). Specific inhibition of bacterial β-glucuronidase by pyrazolo[4,3-*c*]quinoline derivatives via a pH-dependent manner to suppress chemotherapy- induced intestinal toxicity. J. Med. Chem..

[17] Mekheimer R.A. (1999). Fused quinoline heterocycles I. First example of the 2,4-diazidoquinoline-3-carbonitrile and 1-aryl-1,5-dihydro-1,2,3,4,5,6-hexaazaacephenanthrylenes ring systems. J. Chem. Soc. Perkin Trans. 1.

[18] Green L.C., Wagner D.A., Glogowski J., Skipper P.L., Wishnok J.S., Tannenbaum S.R. (1982). Analysis of nitrate, nitrite, and [15N]nitrate in biological fluids. Anal. Biochem..

[19] Alderton W.K., Cooper C.E., Knowles R.G. (2001). Nitric oxide synthases: Structure, function and inhibition. Biochem. J..

[20] Marcocci L., Maguire J.J., Droy-Lefaix M.T., Packer L. (1994). The nitric oxide-scavenging properties of Ginkgo biloba extract EGb 761. Biochem. Biophys. Res. Commun..

[21] Ronchetti D., Impagnatiello F., Guzzetta M., Gasparini L., Borgatti M., Gambari R., Ongini E. (2006). Modulation of iNOS expression by a nitric oxide-realeasing derivatives of the natural antioxidant ferulic acid in activatived RAW 264.7 macrophages. Eur. J. Pharmacol..

[22] Wang G.J., Chen S.M., Chen W.C., Chang Y.M., Lee T.H. (2007). Selective inducible nitric oxide synthase suppression by new bracteanolides from Murdannia bracteata. J. Ethnopharmacol..

[23] Mardini I.A., FitzGerald G.A. (2001). Selective inhibitors of cyclooxygenase-2: A growing class of antiinflammatory drugs. Mol. Interv..

[24] Yang H., Yin P., Shi Z., Ma Y., Zhao C., Zheng J., Chen T. (2016). Sinomenine, a COX-2 inhibitor, induces cell cycle arrest and inhibits growth of human coloncarcinoma cells in vitro and in vivo. Oncol. Lett..

[25] Halawany A.M.E., Sayed N.S.E., Abdallah H.M., Dine R.S.E. (2017). Protective effects of gingerol on streptozotocin-induced sporadic Alzheimer’s disease: Emphasis on inhibition of β-amyloid, COX-2, alpha-, beta-secretases and APH1a. Sci. Rep..

[26] Li Y., Tian D., Zhu C., Ren L. (2016). Demethoxycurcumin preserves renovascular function by downregulating COX-2 expression in hypertension. Oxid. Med. Cell. Longev..

[27] Tung C.W. (2013). Prediction of pupylation sites using the composition of k-spaced amino acid pairs. J. Theor. Biol..

[28] Tung C.W., Wu M.T., Chen Y.K., Wu C.C., Chen W.C., Li H.P., Chou S.H., Wu D.C., Wu I.C. (2013). Identification of biomarkers for esophageal squamous cell carcinoma using feature selection and decision tree methods. Sci. World J..

[29] Tseng C.H., Tung C.W., Wu C.H., Tzeng C.C., Chen Y.H., Hwang T.L., Chen Y.L. (2017). Discovery of indeno[1,2-*c*]quinoline derivatives as potent dual antituberculosis and anti-inflammatory agents. Molecules.

[30] Gramatica P., Corradi M., Consonni V. (2000). Modelling and prediction of soil sorption coefficients of non-ionic organic pesticides by molecular descriptors. Chemosphere.

[31] Hall L.H., Kier L.B. (1995). Electrotopological state indices for atom types: A novel combination of electronic, topological, and valence state information. J. Chem. Inf. Comput. Sci..

[32] Liu R., Sun H., So S.S. (2001). Development of quantitative structure−property relationship models for early ADME evaluation in drug discovery. 2. Blood-brain barrier penetration. J. Chem. Inf. Comput. Sci..

[33] Steinbeck C., Han Y., Kuhn S., Horlacher O., Luttmann E., Willighagen E. (2003). The Chemistry Development Kit (CDK): An open-source Java library for Chemo- and Bioinformatics. J. Chem. Inf. Comput. Sci..

[34] Todeschini R., Consonni V. (2009). Molecular Descriptors for Chemoinformatics.

[35] Roehm N.W., Rodgers G.H., Hatfield S.M., Glasebrook A.L. (1991). An improved colorimetric assay for cell proliferation and viability utilizing the tetrazolium salt XTT. J. Immunol. Methods.

[36] Bradford M.M. (1976). A rapid and sensitive method for the quantitation of microgram quantities of protein utilizing the principle of protein-dye binding. Anal. Biochem..

[37] Yap C.W. (2011). PaDEL-descriptor: An open source software to calculate molecular descriptors and fingerprints. J. Comput. Chem..

