# Supplementary material for: Discovery of Pyrazolo[4,3-c]quinolines Derivatives as Potential Anti-Inflammatory Agents through Inhibiting of NO Production
Source: Molecules. 2018 Apr 28;23(5):1036. doi: 10.3390/molecules23051036 (PMC6102577; doi:10.3390/molecules23051036)

AP-2-OMe-An

Mercury-400BB "Mercuryplus400"
Date: Oct 8 2008
Solvent: DMSO
Ambient temperature
Total 36 repetitions

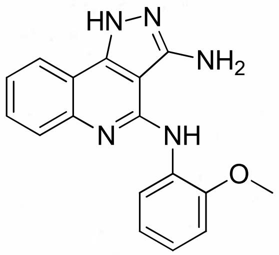

2c

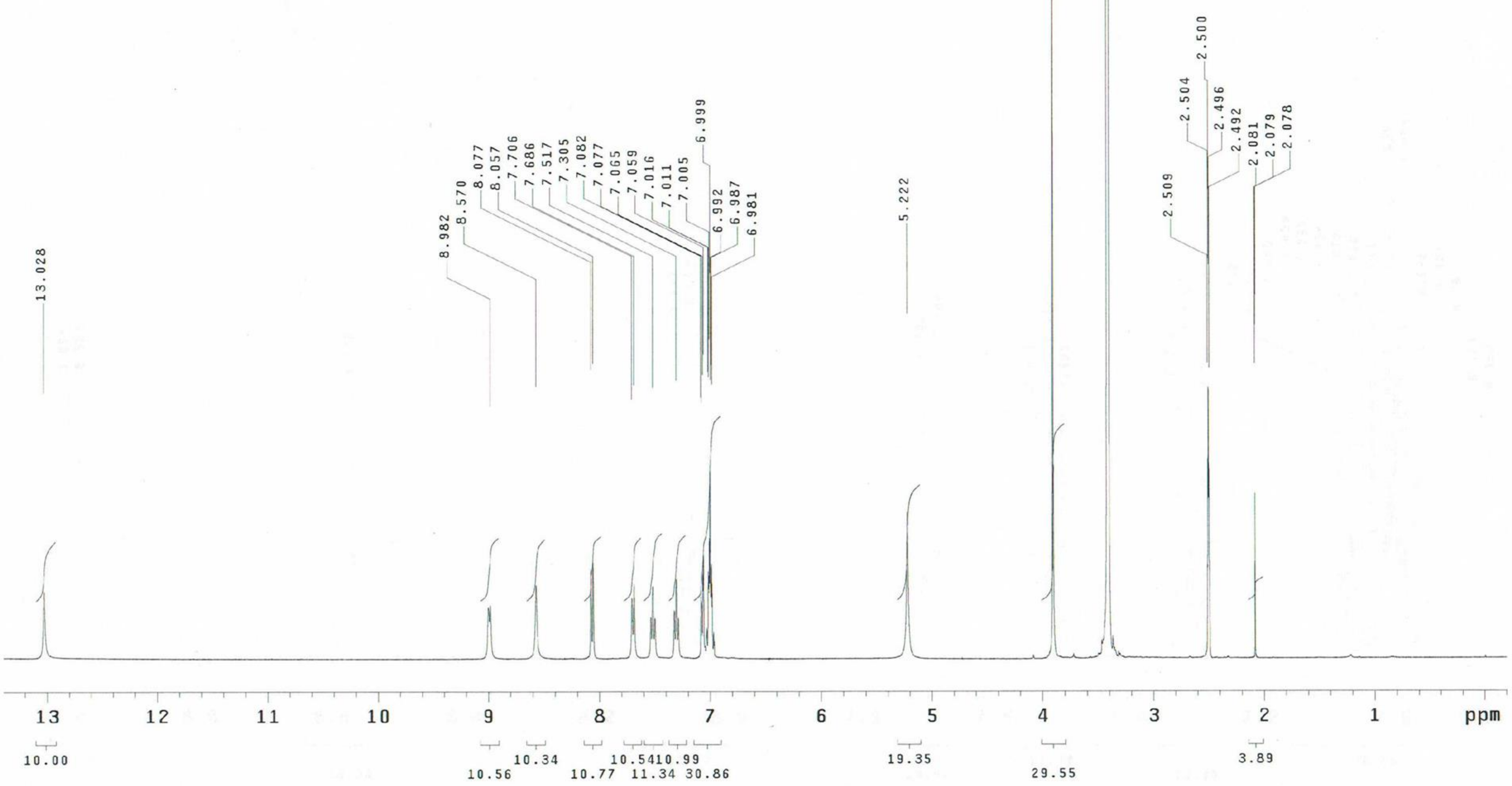

AP-3-OMe-An
Pulse Sequence: s2pul
UNITYplus-400 "unityplus400"
Date: Oct 28 2008
Solvent: DMSO
Ambient temperature
Total 64 repetitions

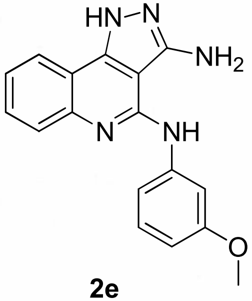

2e

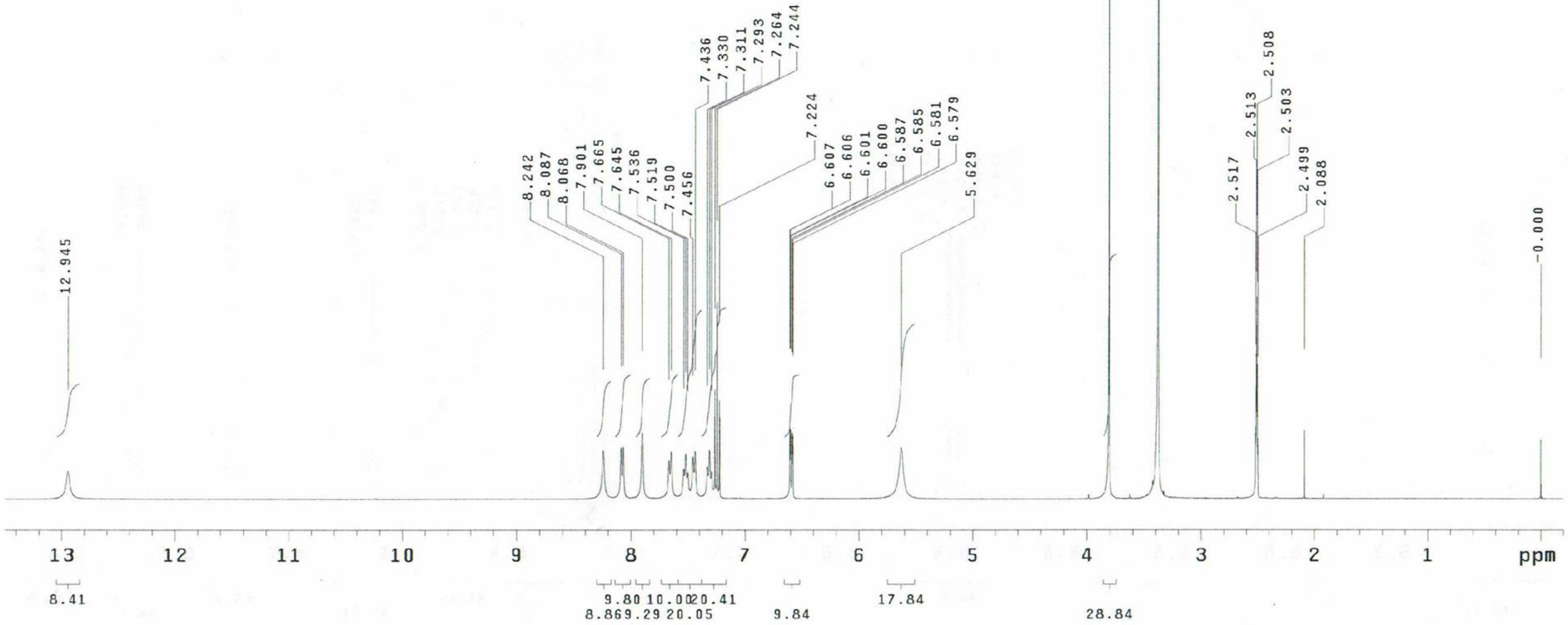

AP-m-COCH3

Pulse Sequence: s2pul
UNITYplus-400 "unityplus400"
Date: Aug 23 2007
Solvent: DMSO
Ambient temperature
Total 64 repetitions

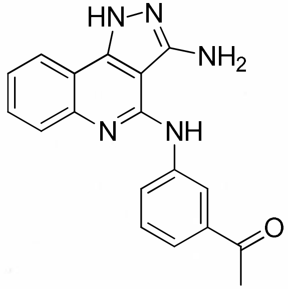

2f

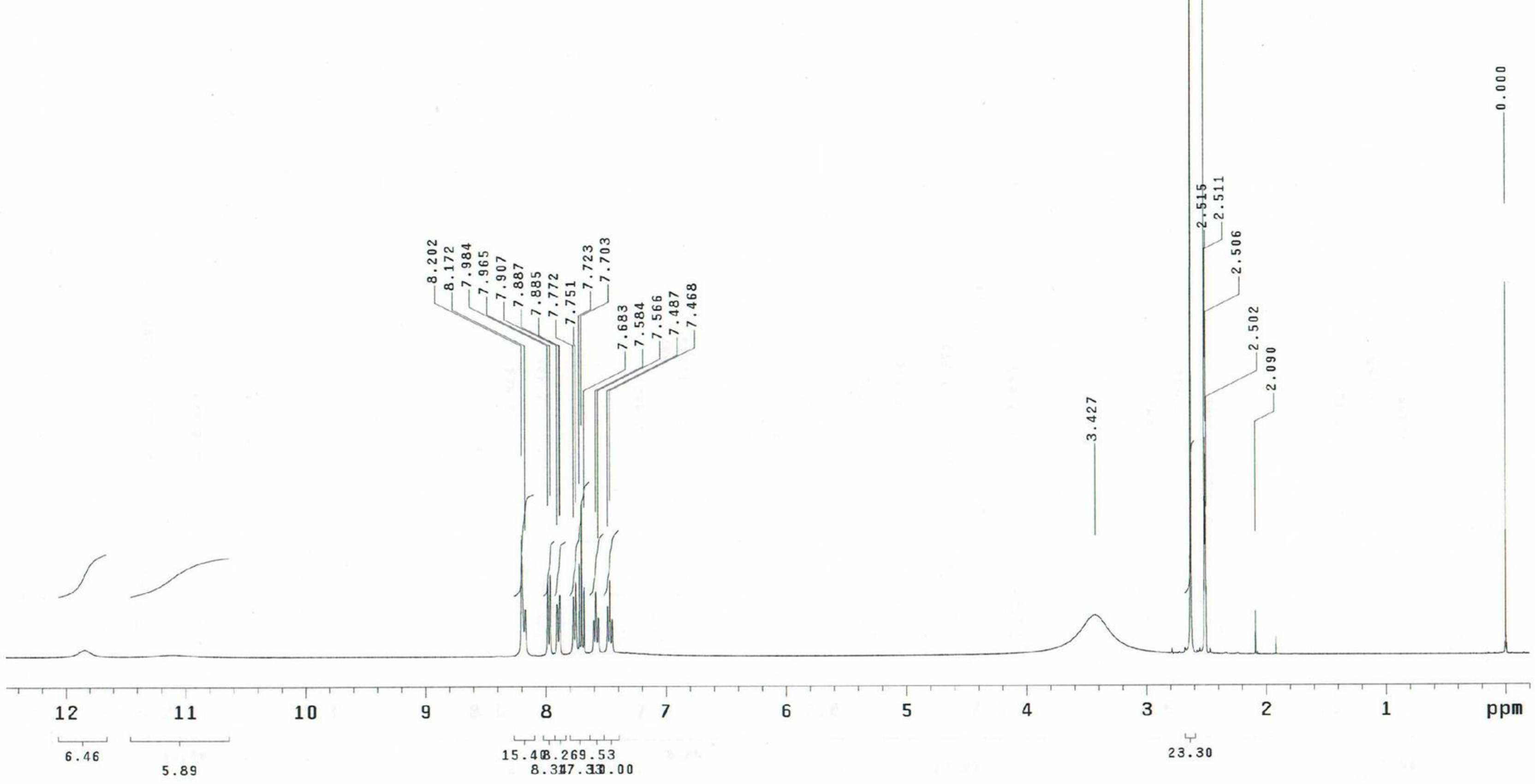

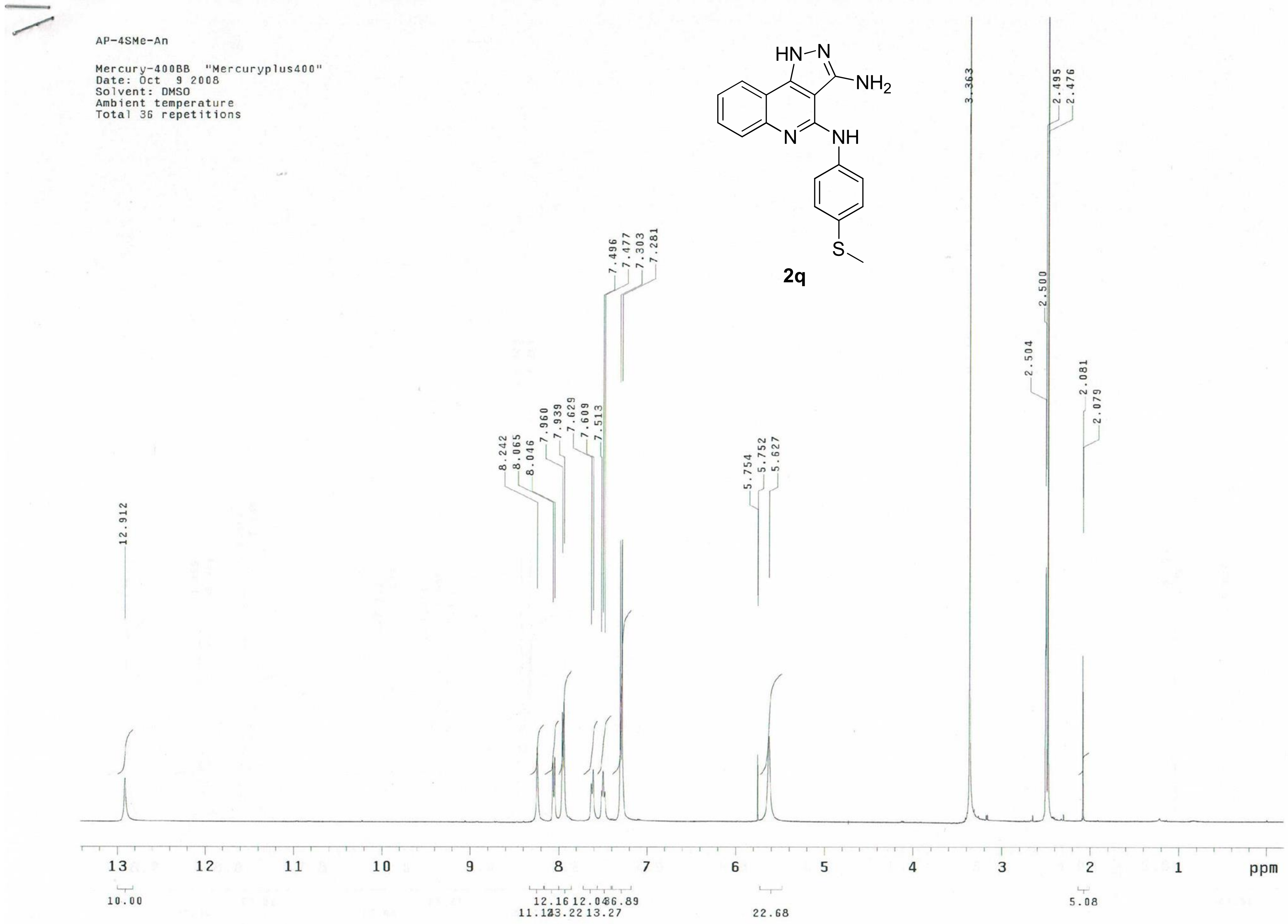
AP-4SMe-An
Mercury-400BB "Mercuryplus400"
Date: Oct 9 2008
Solvent: DMSO
Ambient temperature
Total 36 repetitions
HN-N
NH2
N
NH
S
12.912
8.242
8.065
8.046
7.960
7.939
7.629
7.609
7.513
7.496
7.477
7.303
7.281
5.754
5.752
5.627
3.363
2.504
2.500
2.495
2.476
2.081
2.079
13 12 11 10 9 8 7 6 5 4 3 2 1 ppm
10.00
12.16 12.04 36.89
11.12 13.22 13.27
22.68
5.08

2q

F-triOMe

Pulse Sequence: s2pul
UNITYplus-400 "unityplus400"
Date: Nov 23 2009
Solvent: DMSO
Ambient temperature
Total 80 repetitions

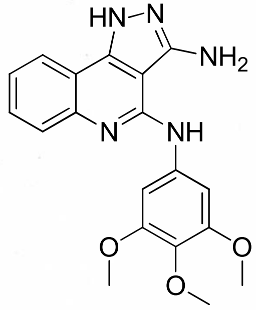

2r

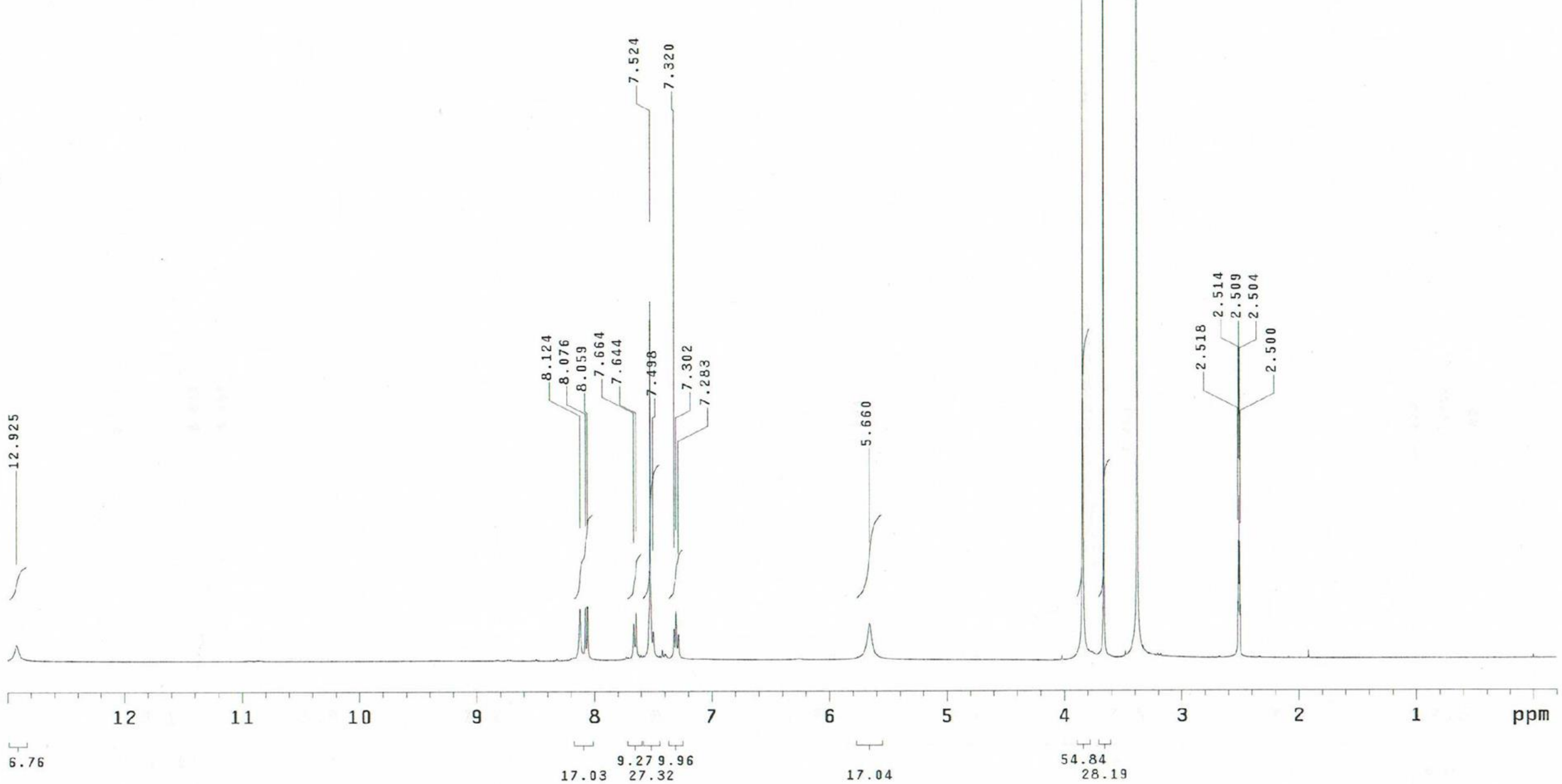

Supplement: Supplementary file 1 [file molecules-23-01036-s001.zip › molecules-298967-supplementary.pdf]
